# Mutations in the Vicinity of the IRAK3 Guanylate Cyclase Center Impact Its Subcellular Localization and Ability to Modulate Inflammatory Signaling in Immortalized Cell Lines

**DOI:** 10.3390/ijms24108572

**Published:** 2023-05-10

**Authors:** Ilona Turek, Trang H. Nguyen, Charles Galea, Isaiah Abad, Lubna Freihat, David T. Manallack, Tony Velkov, Helen Irving

**Affiliations:** 1 Department of Rural Clinical Sciences, La Trobe Rural Health School, La Trobe University, Bendigo, VIC 3552, Australia; hongtrang.nguyen@latrobe.edu.au; 2 La Trobe Institute for Molecular Science, La Trobe University, Bendigo, VIC 3552, Australia; 3 Monash Institute of Pharmaceutical Sciences, Monash University, Melbourne, VIC 3052, Australiadavid.manallack@monash.edu (D.T.M.); 4 Department of Microbiology, Monash University, Wellington Rd, Clayton, VIC 3800, Australia; tony.velkov@monash.edu

**Keywords:** interleukin-1 receptor-associated kinase 3 (IRAK3 or IRAK-M), guanylate cyclase, cyclic guanosine monophosphate (cGMP), human Toll-like receptor 4 (hTLR4), lipopolysaccharide, nuclear factor kappa-light-chain-enhancer of activated B (NFκB), inflammatory cytokines

## Abstract

Interleukin-1 receptor-associated kinase 3 (IRAK3) modulates the magnitude of cellular responses to ligands perceived by interleukin-1 receptors (IL-1Rs) and Toll-like receptors (TLRs), leading to decreases in pro-inflammatory cytokines and suppressed inflammation. The molecular mechanism of IRAK3’s action remains unknown. IRAK3 functions as a guanylate cyclase, and its cGMP product suppresses lipopolysaccharide (LPS)-induced nuclear factor kappa-light-chain-enhancer of activated B cell (NFκB) activity. To understand the implications of this phenomenon, we expanded the structure–function analyses of IRAK3 through site-directed mutagenesis of amino acids known or predicted to impact different activities of IRAK3. We verified the capacity of the mutated IRAK3 variants to generate cGMP in vitro and revealed residues in and in the vicinity of its GC catalytic center that impact the LPS-induced NFκB activity in immortalized cell lines in the absence or presence of an exogenous membrane-permeable cGMP analog. Mutant IRAK3 variants with reduced cGMP generating capacity and differential regulation of NFκB activity influence subcellular localization of IRAK3 in HEK293T cells and fail to rescue IRAK3 function in *IRAK3* knock-out THP-1 monocytes stimulated with LPS unless the cGMP analog is present. Together, our results shed new light on the mechanism by which IRAK3 and its enzymatic product control the downstream signaling, affecting inflammatory responses in immortalized cell lines.

## 1. Introduction

Innate immune responses are the first line in the host’s defense against microbial and endogenous danger signals. The well-controlled and rapid responses are aimed at the elimination of invading microorganisms by establishing an acute inflammatory environment via generation of inflammatory mediators, such as cytokines secreted from immune cells to recruit and activate other cells, initiating a wider immune response for effective clearance of pathogens [1]. This crude but swift reaction to pathogens is possible thanks to the group of evolutionarily conserved pattern-recognition receptors (PRRs) primarily expressed by specialized immune cells (e.g., macrophages, dendritic cells). PRRs act as sensors that recognize conserved molecular patterns carried by microorganisms called pathogen-associated molecular patterns (PAMPs) [2]. PAMPs can vary in their molecular nature and are redundantly or non-redundantly perceived by specific PRRs [2]. Toll-like receptors (TLRs) are a family of PRRs that are either expressed on the cell surface, where they generally detect outer membrane components of bacteria, or intracellularly within endosomal compartments, where they detect foreign (and—in case of autoimmunity—self) nucleic acids.

One of the 10 TLRs identified in humans is TLR4, which, via its extracellular *N*-terminal leucine-repeats domain, binds with high affinity to lipopolysaccharide (LPS)—a proinflammatory molecule found in the outer membrane of most Gram-negative bacteria [3,4], Monomeric molecules of LPS are recognized by a complex of TLR4 and its accessory protein myeloid differentiation 2 (MD2) enabling dimerization of the intracellular Toll interleukin (IL)-1 receptor (TIR) domains of TLR4 monomers and recruitment of other TIR-domain-containing adaptor proteins, such as myeloid differentiation primary response 88 (MyD88), required for further signal transduction [4,5]. MyD88 also contains a death domain that binds to death domains of downstream interleukin-1 receptor-associated kinases (IRAKs), which are indispensable for LPS signal transduction [6], forming a supramolecular organizing center, termed the ‘myddosome’ [7,8,9], allowing subsequent signaling cascades, namely (1) the nuclear factor κ-light-chain-enhancer of activated B cells (NFκB) pathways [10] and (2) the mitogen-activated protein kinase (MAPK) cascade [11,12,13,14], to proceed. Both NFκB (canonical and alternative) pathways are regulated at the level of IRAK modification [15] and all of these pathways result in translocation of NFκB heterodimers from the cytoplasm into the nucleus, where they act as transcription factors by binding to the NFκB target sites in the promoters and enhancers of immune response genes, leading to the induction of numerous inflammatory cytokines, such as interleukin-6 (IL-6) and tumor necrosis factor-α (TNF-α), promoting host defense.

To preserve immune homeostasis and prevent excessive inflammation, which can lead to the development of autoimmune or inflammatory diseases [16], TLR signaling needs to be tightly controlled. Interleukin-1 receptor-associated kinase 3 (IRAK3) was first described as a positive regulator of interleukin-1 receptor (IL-1R)-mediated innate immunity [6], but IRAK3 is now characterized as a negative regulator of TLR signaling, although the molecular mechanism involved in IRAK3-dependent modulation of NFκB activity is poorly defined. IRAK3 prevents dissociation of IRAK1/2 from MyD88 and formation of an IRAK1 complex with tumor necrosis factor receptor-associated factor 6 (TRAF6), contributing to the attenuation of NFκB activation upon TLR4 stimulation with LPS [17,18]. IRAK3 can also directly interact with MyD88–IRAK4 to regulate TLR7-induced mitogen-activated protein kinase (MAPK)/extracellular signal-regulated kinase 1/2 (ERK) kinase kinase 3 (MEKK)-dependent late-phase (after 0.5 h) NFκB activation, exerting inhibition of inflammatory response [19]. Finally, IRAK3 can also negatively regulate the NFκB-inducing kinase (NIK)-IκB kinase α (IKKα)-mediated alternative (non-canonical) pathway, although this mechanism of IRAK3 action was observed in TLR2, but not in TLR4, signaling [20].

In humans, IRAK3 and its three paralogs share similar domain architecture: an N-terminal death domain is followed by the undetermined domain, also referred to as the PST region prone to hyperphosphorylation [21], and the central (pseudo)kinase domain, which (with the exception of IRAK4) is followed by a C-terminal domain containing P-X-E-X-X motif(s) responsible for binding TRAF6 [22,23]. In contrast to the catalytically active kinases IRAK1 and IRAK4, IRAK2 and IRAK3 lack essential active site residues, and although a weak activity was observed for IRAK3 [6], similarly to IRAK2 it is considered a catalytically inactive pseudokinase [6] whose function is largely unknown. Often, pseudokinases develop entirely new enzymatic activities distinct from substrate phosphorylation [24], and such a moonlighting function has recently been identified for IRAK3. Due to the presence of a cryptic guanylyl cyclase (GC) active site buried within its pseudokinase domain, IRAK3 is capable of generating 3′,5′-cyclic guanosine monophosphate (cGMP) [25,26]. The cGMP generated by IRAK3 is implicated in selective modulation of downstream IRAK3-dependent suppression of LPS-induced NFκB activity [26,27]. This is likely via cGMP affecting binding and/or activity of proteins in the immediate vicinity of IRAK3, thus, altering inflammatory signaling cascade(s) through a mechanism that remains poorly understood.

Since IRAK3 functions as a major check-point protein that regulates the extent of innate immune response, its level and/or activity can be modulated to enhance or decrease inflammatory responses. Generally, elevated IRAK3 reduces the ability of an organism to combat secondary infections during endotoxin tolerance [17,28,29], while its under-expression and/or inactivity is associated with chronic inflammation [30,31]. Consequently, IRAK3 was proposed as a useful diagnostic and prognostic marker in inflammation. Induction of IRAK3 expression is required for development of ‘endotoxin tolerance’ [6,17] and ‘cancer-induced monocyte tolerance’ [32], and tumors with high IRAK3 expression show enriched anti-inflammatory pathways and a worse clinical response to immune checkpoint blockade therapy [33]. Since IRAK3 appears to play a pivotal role in attenuating excessive activation of the NFκB and subsequent inflammatory responses, it can be considered a double-edged sword that may be exploited by tumor cells [32] or bacteria [34] to evade active immune surveillance of the host. Intrinsic complexity of signaling pathways modulated by IRAK3 in different cell lines upon receptor stimulation with various ligands accounts for the outcomes of up- or down-regulation of IRAK3 level and activity not being straightforward. Recent studies revealed that TLR stimulation may lead to the suppression of IRAK3 expression. For instance, severe acute respiratory syndrome (SARS-CoV2) decreases IRAK3 expression, thus, rendering macrophages hyper-responsive to TLR stimulation, promoting pro-inflammatory cytokine expression and leading to the cytokine storm [35]. In contrast, suppression of IRAK3 by Blimp-1 in plasmacytoid dendritic cells upon flavivirus infection does not affect IL-6 or TNF-α levels but does accelerate generation of the type I interferon (IFN-1) to limit viral infection [36].

Realization of IRAK3’s potential as a drug target in immunodeficiency diseases and in immunotherapies is hindered by the lack of understanding of its action at a cellular and molecular level. Recent elucidation of the crystal structure of the human IRAK3 pseudokinase domain in a closed, pseudoactive conformation revealed it dimerizes in a unique head-to-head arrangement, leading to further higher-order oligomer formation [37], allowing for additional exploration of functions and mechanistic details of IRAK3, as well as its novel GC activity producing cGMP. Modulating cGMP signaling limits cancer cell proliferation and survival [38,39], and IRAK3 contributes to the progression of both inflammation and cancer, likely via modulation of NFκB signaling, which provides a critical link between both conditions [40,41,42]. Therefore, a deeper understanding of the molecular mechanism(s) by which IRAK3 and cGMP impact the NFκB pathway is needed to enable targeting IRAK3 for therapeutic purposes.

**Figure 1:**
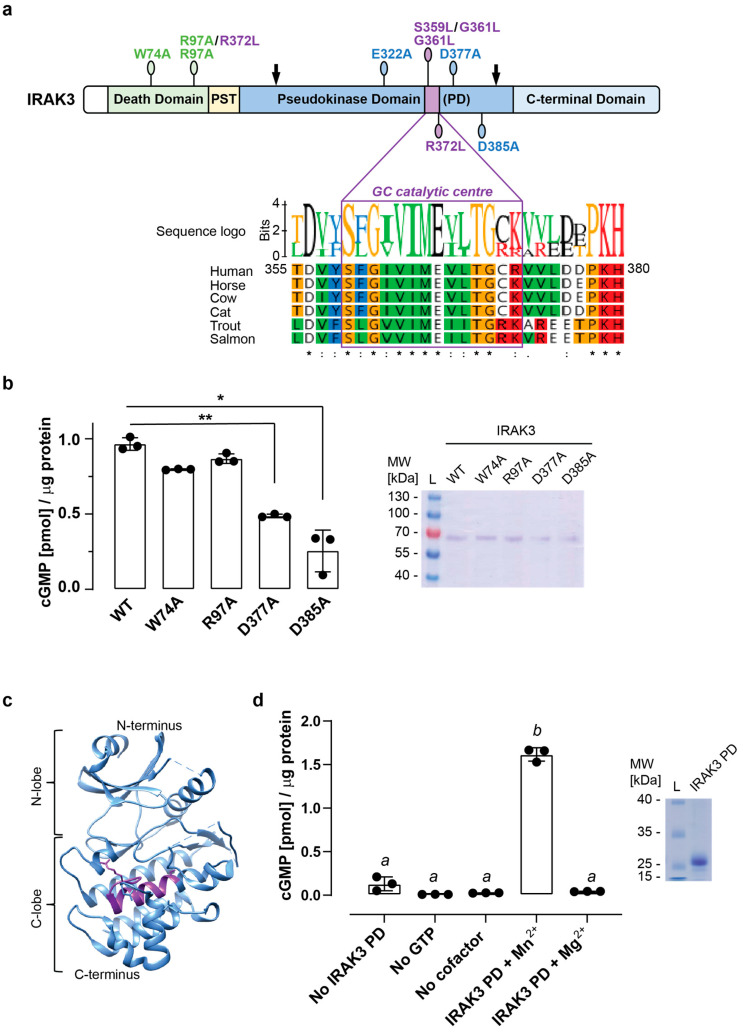
Guanylate cyclase (GC) catalytic center of the IRAK3 pseudokinase domain (PD) generates cGMP in vitro. (**a**) Domain organization of human IRAK3 and multiple sequence alignment of the amino acid residues surrounding and including the GC catalytic center from selected IRAK3 orthologs. Position of mutated amino acids is indicated, and arrows indicate truncated IRAK3 PD (amino acids 201–410). Amino acids are numbered according to human IRAK3 (accession number: Q9Y616) sequence and colored according to the Clustal coloring scheme. Identical residues are indicated with an asterisk (*), conserved residues with a colon (:), and semi-conserved residues with a full stop (.). PST—proline/serine/threonine-rich regions. Sequence logo of the region containing GC center of all available vertebral IRAK3 orthologs is in Supplementary Figure S1a; (**b**) full-length wild type (WT) recombinant IRAK3 and mutated death domain variants generate sub-picomolar amounts of cGMP per µg protein in vitro, while the GC activity of PD mutated variants (D377A, and D385A) is decreased. Results are from three independent experiments (mean ± SD, one-way ANOVA followed by Tukey–Kramer multiple comparison test, *n* = 3, * *p* < 0.05, ** *p* < 0.01; intra-assay CV% = 9.4, inter-assay CV% = 2.4). Inset shows a Coomassie blue-stained SDS-PAGE gel of purified IRAK3 recombinant proteins at the expected molecular weight (MW) of ~68 kDa. L—MW ladder (PageRuler Prestained Protein Ladder, Thermo Fisher Scientific, Waltham, MA, USA). Quantification of cGMP in negative controls is in Supplementary Figure S1d; (**c**) crystal structure of IRAK3 PD (PDB 6RUU), where the GC catalytic center is shown in purple and neighboring amino acids selected for site-directed mutagenesis are shown as stick models. Dotted lines indicate flexible loops without structural information; (**d**) recombinant truncated IRAK3 PD (amino acids 201–410; Supplementary Figure S1e) generates picomolar amounts of cGMP per µg protein in vitro. Different superscript (*a* and *b*) indicates significantly different results from three independent experiments (mean ± SD, one-way ANOVA followed by Tukey–Kramer multiple comparison test, *n* = 3, *p* < 0.0001; intra-assay CV% = 9.5, inter-assay CV% = 6.1). Inset shows a Coomassie brilliant blue-stained SDS-PAGE gel of purified recombinant IRAK3 PD at the expected molecular weight (MW) of ~27 kDa. L—MW ladder (PageRuler). Uncropped SDS-PAGE images are in Supplementary Figure S1b,c.

To fill these knowledge gaps, we examined the contribution of residues located in the vicinity of the GC center of IRAK3 (Figure 1a) on generation of cGMP and the regulation of NFκB signaling. Using site-directed mutagenesis, we interrogated the function of residues located in different domains of IRAK3 and predicted to be of potential importance to its cellular function on the production of cGMP in vitro and in the transiently transfected immortalized cell line HEK293T. Several residues of IRAK3 altered its ability to produce cGMP and, correspondingly, modified the effects of transiently transfected IRAK3 on the downstream LPS-induced NFκB activity, which can be partially restored by supplementation with a membrane-permeable cGMP analog. Our findings shed light on IRAK3 enzymatic activity and the impact of its cGMP product on inflammatory responses mediated by NFκB.

## 2. Results

### 2.1. Mutations in the Vicinity of the Guanylate Cyclase Center Modulate IRAK3 Capacity to Generate cGMP

Mutation of the highly conserved (Figure 1a, Supplementary Figure S1a) positively charged residue (R372L) located in the guanylate cyclase (GC) catalytic center of human IRAK3 has been shown to abolish the ability of the protein to generate cGMP [26]. Therefore, we set out to investigate how other surface-exposed residues predicted or known to be crucial for different aspects of IRAK3 activity affected its GC activity. Using site-directed mutagenesis, we generated point mutations of two amino acids (W74A, R97A) in the death domain region (Figure 1a). A conserved W74 is a key residue for interaction with the IRAK4 death domain [19] and, similarly to R97, is crucial for NFκB modulation and cytokine expression upon PAMP stimulation [19,43]. Other single amino acid mutations were generated in the pseudokinase domain outside of the GC region of IRAK3, where negatively charged E322, D377, and D375 were substituted with alanine (A). Both mutated versions of the IRAK3 GC catalytic center, where single residues were predicted to be of importance for the enzymatic activity of the protein (G361 and R372) [26], were substituted with hydrophobic leucine (L). Finally, we also generated two double mutants—R97A/R373L and S359L/G361L. The former contains a mutation in the death domain and the GC catalytic center, while the latter contains two mutations located in the GC catalytic center at residues, which are predicted to assist in binding the guanosine in the GTP substrate (Figure 1a).

Despite being insoluble and unstable while expressed in insect cell cultures (Supplementary Figure S2), the full-length proteins were expressed as *N*-terminally 6 × His-tagged recombinants in bacteria and were purified (Supplementary Figure S1b). We then verified whether equal amounts of purified wild type, two death domain mutant, and two pseudokinase mutated versions of IRAK3 can generate similar amounts of cGMP in vitro. As shown previously [26], the full-length wild type IRAK3 can produce sub-picomolar levels of cGMP per µg protein (Figure 1b). The death domain mutant variants of IRAK3 W74A and R97A both showed similar GC activity to wild type IRAK3 (Figure 1b). In contrast, both D377A and D385A, the pseudokinase domain mutated versions of IRAK3, led to significantly lower levels of cGMP than the wild type IRAK3 (Figure 1b). These results align with the notion that the death domain of IRAK3 is not required for GC enzymatic activity, whereas the D377 and D385 residues, both located in the C-lobe of IRAK3 pseudokinase domain in the vicinity of its GC catalytic center (Figure 1c), appear to play a role in cGMP generation.

To confirm if the pseudokinase domain of IRAK3 containing the embedded GC catalytic center is sufficient for GC activity, we cloned, expressed, and purified an *N*-terminally 6 × His-tagged wild type IRAK3 containing a truncated pseudokinase domain (IRAK3 PD). This protein is more soluble and stable than the full-length IRAK3, and—similarly to the full-length IRAK3—shows a preference for Mn^2+^ ions as a cofactor in generating sub-picomolar levels of cGMP per µg protein in vitro (Figure 1d, Supplementary Figure S1c–f).

**Figure 2:**
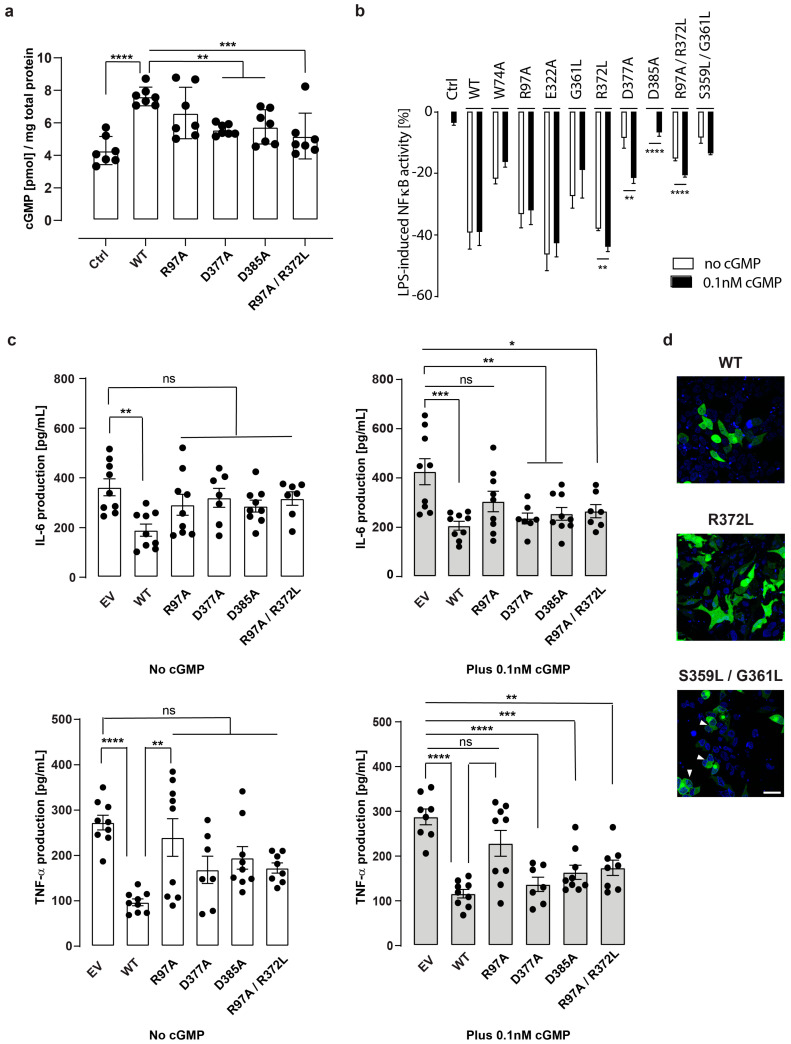
Effect of mutations in IRAK3 protein on cellular processes. (**a**) Enzyme-linked immunoassay quantification of cGMP generated by C-terminally eGFP-tagged wild type (WT) and IRAK3 mutated either in the death domain (R97A), in the region surrounding the GC center (D377A, D385A), or both (R97A/R372L double mutant). HEK293T cells transiently transfected with a plasmid containing IRAK3 gene or with an empty (containing eGFP-encoding gene) vector as a negative control (Ctrl) and induced with Mn^2+^ ions. Data shown as mean ± SD, one-way ANOVA followed by Dunnett’s multiple comparison test, *n* = 7, ** *p* < 0.01, *** *p* < 0.001, **** *p* < 0.0001; intra-assay CV% = 5.4, inter-assay CV% = 8.8); (**b**) sub-nanomolar levels of 8-Br-cGMP alter the effect of IRAK3 GC mutants on LPS-induced NFκB activity in HEK-Blue hTLR4 cells. Cells were transiently transfected with vector harboring only eGFP-encoding gene used as a negative control (Ctrl), eGFP-tagged wild type (WT), or a mutated version of IRAK3. SEAP activity correlating to NFκB activity was measured and normalized to Ctrl 16 h post-induction with 10 ng mL^−1^ LPS plus or minus 0.1 nM 8-Br-cGMP. Data are from three independent experiments (mean ± SEM, unpaired *t*-test with Welch correction, *n* = 9, ** *p* < 0.01, **** *p* < 0.0001; intra-assay CV% = −7.0, inter-assay CV% = −8.5); (**c**) complementation of THP-1 IRAK3^−/−^ knockout cells with IRAK3 (WT, R97A, D377A, D385A, R97A/R372L). THP-1 IRAK3^−/−^ knockout cells were transfected with empty vector (EV, negative control) or vectors carrying wild type or mutated IRAK3 gene. After transfection, the cells were treated with 1 µg/mL LPS with (grey bars) or without (white bars) 0.1 nM 8-Br-cGMP for 24 h, and cell supernatants were collected for TNF-α and IL-6 protein quantification using ELISA (mean ± SEM, *n* = 7–9, one-way ANOVA followed by Turkey’s multiple comparisons test, * *p* < 0.05, ** *p* < 0.01, *** *p* < 0.001, **** *p* < 0.0001, ns: not significant; TNF-α intra-assay CV% = 10.4, inter-assay CV% = 5.8; IL-6 intra-assay CV% = 12.3, inter-assay CV% = 10.0); (**d**) micrographs of Hoechst 33342-stained HEK293T cells, previously transfected with eGFP-tagged IRAK3 constructs, showing differential subcellular localization of the proteins, including nuclear exclusion of the S359L/G361L mutant (arrowheads). Bar = 20 µm. Green fluorescence indicates eGFP-IRAK3 constructs, whereas blue fluorescence indicates Hoechst 33342-stained nuclei.

We also verified the GC activity of the mutated versions of IRAK3 upon transient-transfection of the human embryonic kidney cells (HEK293T) with C-terminally-tagged constructs. Mutation in the death domain of IRAK3 maintained cGMP production similar to the wild type IRAK3. Substitution of D377 and D385, located in the pseudokinase domain, with alanine resulted in decreased GC activity comparable to the enzymatic activity of the R97A/R372L double mutant used as a positive control (Figure 2a).

### 2.2. Mutations in IRAK3 Pseudokinase Domain Modify LPS-Induced NFκB Activity

The ability of the eGFP-tagged wild type IRAK3 to decrease LPS-induced NFκB activity in HEK-Blue hTLR4 cells was first shown by Freihat et al. [26] using the secreted embryonic alkaline phosphatase (SEAP) assay. We confirmed this finding and investigated whether the mutated versions of IRAK3 differentially affect NFκB signaling upon cell stimulation with a low concentration of LPS (Figure 2b). In our experiments, cells were transfected with equivalent amounts of each construct, and data were normalized to the eGFP signal to compensate for differences in transfection efficiency. Interestingly, among the nine mutated versions of IRAK3 investigated, the D385A, D377A, and S359L/G361L mutants showed the least capacity to decrease the LPS-induced NFκB activity, suggesting the importance of these residues in modulating this signaling pathway. Of particular interest, the D385A appeared to completely lose the ability to modulate the LPS-dependent NFκB activity (Figure 2b).

These three mutated versions of IRAK3 demonstrated a reduced capacity to raise cellular cGMP levels compared to wild type IRAK3 or the death domain mutant (Figure 2a), pointing at the positive impact of cGMP on the negative regulation of the LPS-induced NFκB activity. Therefore, we supplemented the cells with a sub-nanomolar concentration of membrane-permeable 8-Br-cGMP. As expected, LPS-induced NFκB activity in cells containing wild type IRAK3 or IRAK3 with mutations in the death domain do not change under these conditions. However, the minute amounts of cGMP enhanced the ability of each of the three mutants in the GC center or its vicinity to decrease the LPS-induced NFκB activity (Figure 2b). This result is in line with the findings of Freihat et al. [26], where the activity of the R372L mutant was restored to the activity levels of the wild type. However, the activities of the D377A, D385A, S359L/G361L, or R97A/R372L were not restored to the wild type IRAK3 activity under the conditions used here (Figure 2b).

### 2.3. Wild Type and Mutant IRAK3 Differentially Affect Cytokine Production

Next, we asked whether the reduction in the ability of these mutated versions of IRAK3 to decrease LPS-induced NFκB activity is accompanied with abnormalities in proinflammatory cytokine production. For that purpose, we used a THP-1 IRAK3^−/−^ knockout system, as monocytic cells abundantly express endogenous IRAK3 [27]. THP-1 IRAK3^−/−^ cell lines (K6-3) were transfected with plasmid carrying a gene encoding eGFP-tagged wild type IRAK3 or equivalent amount of plasmid carrying a mutated gene, resulting in mutations in the death domain (R97A) or in the vicinity of the guanylate cyclase center (D377A or D385A) of IRAK3, or the double mutant R97A/R372L.

Measurement of IL-6 and TNF-α levels following stimulation with LPS revealed that THP-1 IRAK3^−/−^ cells complemented with wild type IRAK3 displayed statistically significant inhibition of these proinflammatory cytokines (Figure 2c). However, none of the mutants were capable of decreasing cytokine levels, compared with the empty vector-transfected cells used as a control (Figure 2c), not only confirming that functional death domain of IRAK3 is necessary for inhibition of cytokine production in TLR4-mediated signaling [43], but also revealing the influences of GC activity of IRAK3 on this process. Moreover, LPS-induced TNF-α levels in the complementation with the IRAK3 wild type were significantly different from the levels for the complementation with the IRAK3 R97A mutant, but not statistically different from other GC mutated versions of IRAK3 (Figure 2c), suggesting the effect of GC center is dispensable for the suppression of TNF-α levels.

When sub-nanomolar membrane-permeable cGMP was added to THP-1 IRAK3^−/−^ cells complemented with IRAK3 wild type or with IRAK3 carrying a single mutation in the guanylate cyclase center (D377A, D385A), or with the double mutated (in the death domain and the guanylate cyclase center) IRAK3 (R97A/R372L), TNF-α and IL-6 levels were significantly suppressed compared with cells transfected with the empty vector (Figure 2c). However, supplementation with sub-nanomolar membrane-permeable cGMP had no effect on TNF-α and IL-6 levels in cells complemented with the death domain mutant (R97A) (Figure 2c). Thus, these findings correlate with those on NFκB activity (Figure 2b), implying that low amounts of cGMP (0.1 nM) can partially recover the inhibitory action of the guanylate cyclase center mutants of IRAK3 on TNF-α and IL-6 production.

### 2.4. Mutations in the Pseudokinase Domain of IRAK3 Alter Its Subcellular Localization

Since IRAK3 is considered mainly as a cytoplasmic protein that under certain conditions, including cell stimulation [44], may undergo nuclear or even mitochondrial transportation [45], we verified the subcellular distribution of the wild type and mutated versions of the C-terminally eGFP-tagged IRAK3 proteins transiently expressed in HEK293T cells. Most constructs showed cytoplasmic and, to a lesser extent, nuclear localization. Notably, a large proportion of cells transiently transfected with the S359L/G361L IRAK3 construct, in which two amino acids predicted to be responsible for formation of a hydrogen bond with the GTP substrate in the GC catalytic center and the substrate specificity, respectively, were mutated to hydrophobic leucine residue, displayed nuclear exclusion of the protein (Figure 2d). This suggests that differential subcellular localization of mutated versions of IRAK3 may not only be directly dependent on its interactions with other proteins, but also on the cGMP it generates.

### 2.5. Negatively Charged Patch in the Conserved Region of IRAK3 C-Lobe May Be Required for Cofactor Binding and Is Predicted to Mediate Protein-Protein Interactions

**Figure 3:**
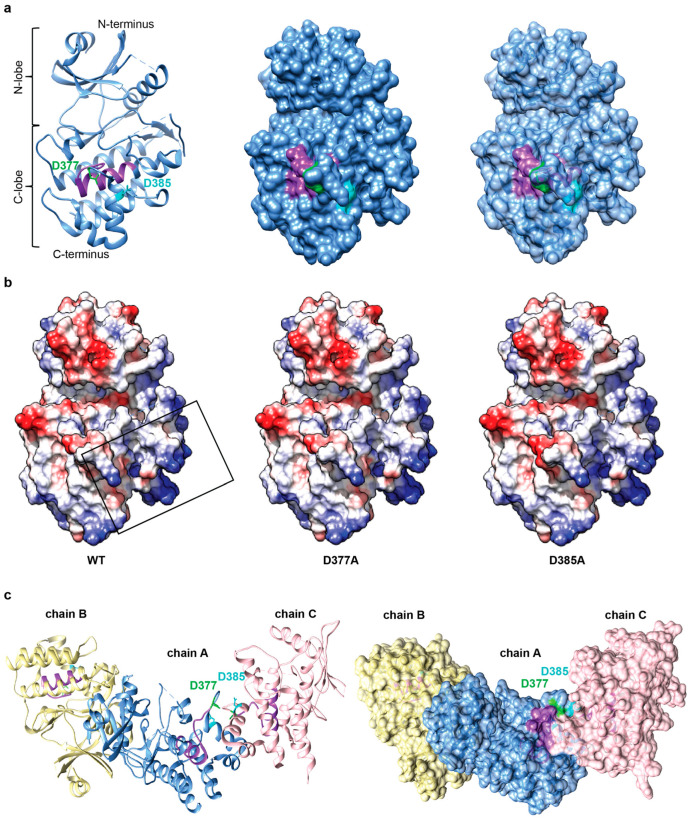
Abolishment of the negative charge on selected residues surrounding IRAK3 guanylyl cyclase (GC) catalytic center may negatively affect its oligomerization. (**a**) Ribbon (**left**), surface (**middle**), and semi-transparent surface (**right**) model of IRAK3 pseudokinase domain (PD) with residues D377 (green) and D385 (cyan) located in the C-lobe and predicted to participate in metal ion cofactor binding, are shown as stick models. Guanylyl cyclase catalytic center is shown in purple; (**b**) electrostatic surface potentials of the wild type (WT) and mutated versions of IRAK3 (D377A and D385A). D377 and D385 are located on conserved surface at IRAK3 C-lobe (square). Coulombic coloring (negative and positive potential indicated with red and blue, respectively) was applied; (**c**) residues D377 and D385 of IRAK3 are predicted to participate in formation of IRAK3 homo-oligomers. Cartoon representations of three IRAK3 PD molecules in the asymmetric unit (chains A–C) that forms a hexamer with three symmetry-related molecules [37]. D377 and D385 (green and cyan, respectively) located in αG-to-αG interface are shown as stick representation in a ribbon (**left**) and a semi-transparent surface (**right**) model. GC catalytic center is shown in purple.

IRAK3 with mutations in the vicinity of the GC catalytic center is less capable of generating cGMP in vitro and in transiently transfected immortalized cell lines than the wild type protein (Figure 1b and Figure 2a). The D377A is located in a loop between αF and αG, and D385A is positioned in αG [37] (Figure 3a). However, docking of the GTP substrate to the IRAK3 pseudokinase domain model reveals that neither D377 nor D385 is involved in the interaction. Instead, the guanine points into the GC cavity towards the first residues of the GC catalytic center, while the phosphate is positioned outwards, near the positively charged R372 residue (Supplementary Figure S1g). Nevertheless, since these residues are conserved among IRAK3 orthologs and are located in the conserved region of the IRAK3 C-lobe (Figure 3b), it is likely that these negatively charged residues may bind the cofactor Mn^2+^ ions. Abolishing the negative charge of D377 or D385 by substitution with alanine (A) significantly altered the electrostatic surface potential of IRAK3 (Figure 3b), which could also have implications on the binding of IRAK3 interactors, including itself. Although according to the recently crystalized IRAK3 pseudokinase domain structure neither D377 nor D385 participate in the ‘head-to-head’ homodimer formation (A-B/A’-B’), it is still likely that mutations of these residues result in disturbed amino acid interactions between chain A (A’) and chain C (C’) of IRAK3 pseudokinase domain molecules in the asymmetric unit (Figure 3c), so that a higher-order helical hexamer (B-A-C-C’-A’-B’), or even a trimer (B-A-C), may not assemble [37]. Moreover, these changes may hinder interactions with other proteins.

## 3. Discussion

Ever since the identification of IRAK3 as a novel GC [25], questions remained concerning the impact of its catalytic activity and the cGMP product itself on the subcellular localization, interactions, and downstream signaling. Changes in some of the processes modulated by IRAK3, such as differences in LPS-induced NFκB activity, have previously been linked to the malfunction of the GC activity of IRAK3 [26].

IRAK3 is one of the proteins crucial for striking a balance between a deficient initial response to a pathogen and an excessive one. Until a thorough understanding of the underlying biochemical mechanisms of its action is obtained, effective targeting of IRAK3 for therapeutic purposes remains a challenging task. Despite IRAK3 acting as an inhibitor of inflammation, it also drives both NFκB and transcription of inflammatory mediators [6,19,43]. This paradoxical function of IRAK3 is not fully understood but is believed to derive from the ability of IRAK3 to induce the transcription of inflammatory inhibitors via MEKK3-dependent NFκB by interacting with IRAK4 [19]. This results in ‘switching’ the pro-inflammatory NFκB signals, induced by IRAK4 via IRAK1/2, to anti-inflammatory signals. Furthermore, an elegant single-cell study revealed that NFκB oscillatory dynamics involves an autoinhibitory loop of IRAK1 and depends on the nature of stimulation; higher levels of LPS decrease NFκB oscillations, shorten response, and render cells insensitive to further stimuli that may prevent the harmful effects of enhanced inflammation [46]. Plausibly discrepancies in IRAK3-dependent modulation of NFκB activity observed in the literature may result from the dose-dependent inhibition of signal flows occurring during TLR and IL-1R signaling, in which low ligand concentrations induce signaling dependent on IRAK1 abundance, while high concentrations trigger IRAK1 autoinhibition, halting further signaling [46]. IRAK3’s importance in clinical tolerance is well established, but whether IRAK3 may also act as a dose-sensing node or is involved in these processes is still to be investigated. The discrepancies in augmentative and repressive functions of IRAK3 on the NFκB activity may be attributed to differing concentrations and types of LPS, the different timepoints considered, type of measurement, as well as differences in the IRAK3 constructs and cell lines used [28].

The ability of IRAK3 to spontaneously enhance NFκB activation depends on its death domain where W74 residue is crucial for basal (spontaneous, non-stimulated) NFκB activation [19,43] and is suggested to be involved in the interaction with IRAK4 [19] or IRAK2 tetramers, as well as being involved in the inhibitory actions of IRAK3 on cytokine production [43]. Located on the other side of the death domain, R97 residue is implicated in binding to the site of the IRAK4 tetramer and also of importance for stimulation of the basal NFκB activation [43]. Importantly, both these interfaces were implicated in activating different pathways, with the former suggested to participate in modulation of IL-8 transcription, while the latter was speculated to predominantly affect IL-8 translation upon overexpression in 293T cells [43]. Under the conditions used in our study, only the mutation of W74, but not R97, resulted in reduced ability of the mutant, compared with the wild type IRAK3, to significantly decrease the LPS-induced NFκB activity in HEK-Blue hTLR4 cells (Figure 2b). Supplementation of the cells with a membrane-permeable cGMP analog did not affect this ability (Figure 2b), in agreement with the capacity of the death domain mutants (W74A and R97A) to generate cGMP levels comparable to the wild type IRAK3 in vitro and in the transiently transfected immortalized cell line (Figure 1b and Figure 2a).

Although any potential modulatory function of the death domain on cGMP generation by IRAK3 (and vice versa) cannot be ruled out, these results are suggestive of the dispensability of the death domain for the GC enzymatic activity of IRAK3. Indeed, a truncated version of IRAK3 containing a portion of its pseudokinase domain, where the GC catalytic center has initially been predicted (Figure 1a) [25], was sufficient to generate comparable quantities of cGMP to the amount produced by the full-length IRAK3 in vitro (Figure 1b,d). Similarly to the R372 residue in the GC catalytic center of IRAK3 being crucial to the generation of cGMP [26], mutation of single residues located in the vicinity of the GC center and predicted to bind its manganese cofactor can substantially decrease enzymatic activity of IRAK3 and its ability to reduce LPS-induced NFκB activity. In particular, IRAK3 D377A and D385A mutations not only showed decreased GC activity compared with the wild type in vitro and in HEK293T (Supplementary Figure S1b and Figure 2a), but also diminished or completely failed to reduce NFκB activity upon LPS treatment (Figure 2b). Although neither D377 nor D385 are predicted to be directly involved in binding the GTP substrate to IRAK3 (Supplementary Figure S1g), both mutations partially regained the ability to decrease LPS-induced NFκB activity upon supplementation with a sub-nanomolar concentration of membrane-permeable cGMP analog (Figure 2b) reminiscent of the R372L mutant [26] and confirmed with the R97A/R372L mutant (Figure 2b).

The guanylate cyclase mutants D377A and D385A and double mutant R97A/R372L also attenuated inflammatory cytokine (TNF-α, IL-6) release (Figure 2c). The less efficient reduction in TNF-α and IL-6 levels correlates with decreased guanylate cyclase activity by these mutants, since the cGMP supplement rescued the inflammatory inhibition of D377A, D385A, and R97A/R372L mutants, but not the R97A mutant (Figure 2c). The association of cGMP with IRAK3 actions was previously shown by the depletion of cGMP effect on inflammation in IRAK3 knockdown monocytes, compared to IRAK3 wild type monocytes where the same levels of cGMP reduced LPS-induced TNF-α and IL-6 levels [27]. However, GC activity of IRAK3 appeared to be dispensable for cytokine production, as inflammatory cytokine levels in the complementation with GC mutants were lower than with the death domain mutant (R97A) (Figure 2c). The observation that sub-nanomolar cGMP supplement had no effect on the death domain mutant, while affecting the guanylate cyclase mutants (Figure 2c), highlights that low amounts of cGMP partially recover the inhibitory action of IRAK3 GC mutants on TNF-α and IL-6 production upon LPS treatment. However, a limitation to the study is that these experiments were only performed using transiently transfected immortalized cell lines. It would be of interest to verify the physiological significance of the mutations within IRAK3 protein in primary cell lines derived from individuals of different ages and sexes.

Of note, both D377 and D385 residues are located in a conserved negative C-lobe patch of IRAK3 (Figure 3a,b) and may contribute to IRAK3 homo-oligomerization (Figure 3c). Weak interaction of the IRAK3 pseudokinase domain with phosphorylated IRAK4 has been shown in vitro, and models of putative IRAK3-IRAK4 hetero-oligomerization have been proposed [37]. D385 is located at a predicted C-lobal interface of the IRAK3–IRAK4 dimer [37], and, thus, it is tempting to speculate that the IRAK3 pseudokinase domain, and possibly the product of its GC activity, modulates not only IRAK3 pseudokinase domain oligomerization (Figure 3c), but also the kinase activity of IRAK4. Such specialized forms of kinase regulation by homo- and heterodimerization with other (pseudo)kinases has been observed in several kinases [24,47,48,49,50]. Abolishment of this negative residue at position 385 may alter interactions between these proteins and have implications on their catalytic activities. It is also possible that these two mutant variants may not only alter protein–protein interactions of IRAK3 (e.g., with IRAK4), but also affect other processes (e.g., post-translational modifications), which are crucial in driving the LPS-dependent NFκB signaling. The significantly compromised ability of the D377A and D385A mutant variants of IRAK3 to decrease the LPS-induced NFκB activity could be partially restored in the presence of sub-nanomolar concentrations of cGMP (Figure 2b), and cyclic nucleotides affect post-translational modifications, including phosphorylation and ubiquitination.

The IRAK3 S359L/G361L double mutation in the GC catalytic center where GTP binding is predicted to be significantly affected (Supplementary Figure S1g) also failed to decrease LPS-induced NFκB activity (Figure 2b). Furthermore, a large proportion of eGFP-tagged S359L/G361L IRAK3 mutant was excluded from the nucleus in transiently transfected HEK293T cells (Figure 2d) compared with predominantly cytoplasmic and nuclear localization of wild type and other IRAK3 mutations. In resting macrophages, a significant proportion of IRAK3 was located near the plasma membrane, bound to detergent-resistant membranes [51]; interestingly, the addition of polymorphonuclear neutrophils to THP-1 macrophages expressing IRAK3 resulted in redistribution of IRAK3 and CASP-6 to the perinuclear space [51]. Altered post-translational modifications may contribute to differential subcellular localization of the IRAK3 mutant(s).

IRAK3 undergoes nuclear export upon challenge with TLR2 ligand Pam_3_CSK_4_ of undifferentiated human promonocytic THP-1 or murine bone marrow-derived macrophages (BMDM) cells [52]. Although it is unclear which proteins participate in modulating IRAK3 subcellular localization, IRAK3 translocates to the nucleus upon activation of PIN1, which catalyzes cis–trans isomerization of IRAK3 phosphorylated by IRAK1 at position S110, with a pS110-P motif serving as an interaction site with PIN1 [44]. Direct binding of PIN1 to IRAK3 in dendritic DC 2.4 cell challenged with IL-33 (but not LPS) requires the death domain of IRAK3 and impacts IRAK3 conformation and dissociation from the myddosome, enhancing IRAK3 stability and its shuttling to the nucleus [44]. Even though the function of nuclear-localized IRAK3 and the reasons for its exclusion from the nucleus are still enigmatic, it is of considerable interest due to the fact that IRAK3 has been shown to regulate chromatin remodeling leading to epigenetic silencing of cytokine gene expression in lung macrophages in systemic sepsis [53,54]. Together, our findings extend the mechanistic insight associated with IRAK3 function and warrant further evaluation of IRAK3 potential as a molecular switch and target for intervention.

## 4. Materials and Methods

### 4.1. Amino Acid Sequence Alignment

Amino acid sequences of vertebral IRAK3 orthologs were retrieved from NCBI with pBLAST search using the human IRAK3 sequence (accession number: Q9Y616) as a query. The alignment was performed with ClustalW [55], and sequence conservations of selected IRAK3 orthologs in vertebrates were visualized with WebLogo [56] using Geneious software 2021.2 (https://www.geneious.com/ (accessed on 27 July 2021)).

### 4.2. Generation of the Truncated IRAK3 (IRAK3 PD) and Mutated Full-Length IRAK3 Constructs Using Gateway Recombination Cloning Technology

Cloning of the wild type full-length IRAK3 into pDEST17 (Thermo Fisher Scientific) for expression in bacteria or pcDNA6.2/C-EmGFP-DEST for expression in mammalian cells, and site-directed mutagenesis to introduce mutations (W74A, R97A, E322A, G361L, R372L, D377A, D385A, R97A/R372L, S359L/G361L), were performed as described previously [56]. Correct sequences of the constructs were confirmed by sequencing (Micromon Monash University) with respective primers (Supplementary Table S1). The recombinant IRAK3 construct containing truncated pseudokinase domain (IRAK3 PD; amino acids 201–410) was PCR-amplified from the template full-length IRAK3 construct specific primers (IRAK3 PD-For and IRAK3 PD-Rev, Supplementary Table S1), cloned into pDONR207 (Thermo Fisher Scientific) using Gateway BP Clonase II Enzyme Mix (Thermo Fisher Scientific), and transformed into One Shot Max Efficiency DH5α-T1R (Thermo Fisher Scientific) chemically competent *E. coli* cells via heat shock. Bacteria were grown on LB agar plates or in liquid medium containing 15 µg mL^−^^1^ gentamicin. Plasmids from separate clones were purified using a PureLink Quick Plasmid Miniprep Kit (Thermo Fisher Scientific), and DNA was quantified with a Nanodrop ND-1000 Spectrophotometer (Thermo Fisher Scientific). Plasmids were sequenced with 201-For and IRAK3-m-For primers (Supplementary Table S1) and the plasmid with correct in-frame insertion was recombined into pDEST17 (Thermo Fisher Scientific) with LR Clonase II (Thermo Fisher Scientific) followed by transformation of Library Efficiency DH5α (Thermo Fisher Scientific) chemically competent Escherichia coli cells via heat shock. Bacteria were grown on LB agar plates or in liquid medium containing 100 µg mL^−^^1^ ampicillin, plasmids from separate clones were purified as described above, and were used for bacterial protein expression.

### 4.3. Expression and Purification of Recombinant Proteins in Bacteria

Here, *E. coli* BL21-AI chemically competent cells (Thermo Fisher Scientific) were transfected with 10 ng of pDEST17 plasmid (Thermo Fisher Scientific) containing full-length wild-type or mutated version of IRAK3 construct, or only containing its truncated pseudokinase domain, and positive transformants were selected with 50 μg mL^−^^1^ carbenicillin. Bacterial liquid cultures at OD600 of ~0.4 were induced with 0.2% (*w*/*v*) L-arabinose (Merck) for 4 h at 220 rpm at 26 °C. Cells were harvested by centrifugation, and N-terminally 6 × His-tagged IRAK3 proteins were purified by affinity chromatography using the Ni-NTA agarose (Qiagen) in the presence of the cOmplete EDTA-free protease inhibitor cocktail (Merck) and 1 mM phenylmethylsulfonyl fluoride (PMSF, Merck) at room temperature under native conditions according to the protocol 12 of the QIAExpressionist manual (Qiagen). Buffer exchange (50 mM Tris-HCl, pH 8.0, with 1 mM PMSF and cOmplete EDTA-free protease inhibitor cocktail) and concentration of the eluted proteins was carried out using Amicon Ultra-15 (10,000 or 5000 NMWL cut-off for full-length proteins and truncated IRAK3 PD, respectively) centrifugal filter units (Millipore). Protein concentration was determined by measuring absorbance of protein preparation at λ_280_ using a Nanodrop ND-1000 spectrophotometer. The purity of preparations was verified on SDS-PAGE using 10% separating and 4% stacking gels, run for 1.5 h at 120 V (BioRAD mini electrophoresis setup), stained with Coomassie brilliant blue (Bio-Rad), and then de-stained with 40% (*v*/*v*) methanol, 10% (*v*/*v*) acetic acid, and 50% (*v*/*v*) distilled water.

### 4.4. Detection and Quantification of cGMP Generated In Vitro

The guanylate cyclase activity of the purified 6 × His-tagged wild-type or mutated versions of IRAK3 protein was measured in vitro by incubating 1 μg of the protein in 50 mM Tris-HCl, pH 8.0, with 1 mM PMSF and cOmplete EDTA-free protease inhibitor cocktail, 5 mM MgCl_2_ or 5 mM MnCl_2_, and 1 mM GTP, in a final volume of 100 μL. Background cGMP levels were measured in a mixture that contained the incubation medium but no protein as well as in mixtures containing all combinations of the reaction components with the exception of one ingredient at a time (negative controls). Reactions were incubated for 20 min at room temperature (20 °C) and terminated by boiling for 3 min, cooling the tubes on ice for 2 min, followed by centrifugation at 2300× *g* for 3 min. The resulting clarified supernatant was retained and assayed for cGMP content using the cGMP EIA Biotrak System (RPN226, Amersham Cytiva) following the acetylation protocol according to the manufacturer’s recommendations. Spectrophotometric measurements were performed at λ_405_ using the CLARIOstar Plus (BMG Labtech) and data from three independent experiments were analyzed (*p* value < 0.05; one-way ANOVA followed by the Tukey–Kramer multiple comparison test).

### 4.5. Protein Expression in the Baculovirus/Insect Cell Expression System

A small-scale baculovirus/insect cell expression screen of the wild type IRAK3 and its mutated constructs containing 10 × His-tagged followed by a thrombin protease cleavage site was undertaken by the University of Queensland Protein Expression Facility (PEF). Insect cells Spodoptera frugiperda (Sf9) and Trichoplusia ni (High Five) were grown in ESP 921 insect cell culture medium. pFastBac1 carrying the gene of interest was transformed into DH10EmBacY *E. coli* and plated onto selective LB media containing 50 µg mL^−^^1^ kanamycin, 7 µg mL^−^^1^ gentamicin, 10 µg mL^−^^1^ tetracycline, 100 µg mL^−^^1^ X-gal, and 40 µg mL^−^^1^ isopropyl ß-D-1-thiogalactopyranoside (IPTG). Positive (white) phenotypic colonies were selected for bacmid isolation and PCR analysis. A total of 200 ng of bacmid DNA and 1 µg of TransIT-insect transfection reagent (Mirus) was used for transfection into 6 × 10^5^ adherent Sf9 cells, which were grown in 400 µL of ESF 921 in a 24-well tissue culture plate. The transfected cells were incubated for 7 days at 27 °C. The culture medium containing the recombinant virus (P1) was harvested and used as seed virus stock for further amplification of the recombinant baculovirus in a 24-deep well plate format. Sf9 cells were seeded at 2 × 10^6^ cells mL^−^^1^ in ESF 921 at a volume of 5 mL per well. An appropriate volume of the recombinant P1 virus seed stock was added. Cell density, viability, and diameter were monitored, and the culture supernatant was harvested (P2) by centrifugation when the cells appeared well infected with the virus. A small-scale expression screen was set up in a 24-well plate format using Sf9 or High Five cells. Briefly, 5 mL of insect cells were seeded per well at mid-log phase in ESF 921 and infected with high titer P2 recombinant baculovirus. Cell density, viability, and diameter were monitored for signs of infection. Cells were grown at 27 °C or 21 °C and harvested 48–72 h or 96–120 h post-inoculation, respectively, for analysis. Cell pellets were resuspended in 1 mL lysis buffer (20 mM sodium phosphate, 500 mM sodium chloride, 20 mM imidazole, 1% Triton X-100, cOmplete protease inhibitor tablet, pH 8 at 25 °C). Samples were lysed using a Branson Sonifier in ice for 3 × 10 s (output: 25%). The lysates were centrifuged at 20,000× *g* for 15 min at 4 °C to obtain the soluble fractions. Total lysates and soluble fractions were analyzed. Samples were loaded on 4–12% Bis-Tris SDS-PAGE gels and ran under denatured and reduced conditions. For Western blotting, the gel was transferred onto a PVDF membrane and probed with an anti-His–HRP conjugate antibody (Miltenyi Biotec) at a dilution of 1:5000 for 1 h. Analysis was performed using a Bio-Rad Chemi-Doc XRS+ imaging system.

### 4.6. Mammalian Cell Culture, Transient Transfection and Imaging

Human embryonic kidney 293T (HEK293T) cells (American Type Culture Collection ATCC: CRL-11268) and HEK-Blue hTLR4 cells (InvivoGen, San Diego, CA, USA) were grown in Dulbecco’s modified Eagle’s medium (DMEM; Gibco) with 8% (*v*/*v*) fetal bovine serum (FBS; Gibco), while human monocyte (THP-1) cells (ATCC: TIB-202) were cultured in Roswell Park Memorial Institute (RPMI) 1640 medium (Gibco) with 10% (*v*/*v*) FBS (Gibco), at 37 °C in 5% CO_2_ tissue culture incubator. All cells were tested for mycoplasma before use (at passage 3 once taken from liquid nitrogen) and on a regular basis (until a maximum passage of 20) using the PCR mycoplasma detection kit (TOKU-E). HEK293T and HEK-Blue hTLR4 cells were split twice a week at ~80% confluency, as described previously^26^ using TrypLE Express enzyme (Thermo Fisher Scientific) for cell detachment, while non-adherent THP-1 cells were passaged by removing approximately 80% (*v*/*v*) of medium in the flask and then back-filling with the same volume of fresh medium. Cell viability was determined by the addition of Trypan Blue solution (Merck) and immediate quantification was performed using the Countess II FL automated cell counter (Thermo Fisher Scientific). Cells were transferred to new flasks at a density of ~2.5 × 10^5^ cells mL^−^^1^, and, in the case of HEK-Blue hTLR4 cells, HEK-BLUE selection antibiotics (InvivoGen) were included for every alternate passage.

HEK293T or HEK-Blue hTLR4 cells were transfected with pcDNA6.2/C-EmGFP-DEST plasmid carrying a gene encoding the wild type or mutated version of the IRAK3 protein using Lipofectamine 3000 reagent (Thermo Fisher Scientific) diluted in Opti-MEM reduced serum medium (Thermo Fisher Scientific) following the manufacturers protocol. Briefly, cells were seeded at density of ~1 × 10^5^ cells mL^−1^ in 24-well Corning Costar cell culture plate in 0.5 mL DMEM medium supplemented with 8% (*v*/*v*) FBS per well one day before the transfection. Cells were transfected the following day when their confluence reached ~70%, using the total 50 μL DNA–lipid complex containing 0.5 μg DNA and 1.5 μL Lipofectamine 3000 reagent per well. HEK293T and HEK-Blue hTLR4 cells transfected with an empty pcDNA6.2/C-EmGFP-DEST plasmid, resulting in expression of eGFP protein, or mock-transfected cells were used as controls.

Transfection efficiency was monitored 24 h post-transfection by visualizing the green fluorescence of the reporter emerald eGFP attached to the C-terminus of the WT, and mutated versions of IRAK3 protein and images were taken with a Olympus CKCx5 fluorescence microscope with a fluorescein isothiocyanate (FITC) filter. As controls, HEK293T and HEK-Blue hTLR4 cells were mock-transfected or transfected with an empty pcDNA6.2/C-EmGFP-DEST plasmid, resulting in expression of eGFP protein only. When indicated, HEK293T cells were stained with nuclear-specific Hoechst 33,342 stain (Thermo Fisher Scientific) at 1 µg mL^−1^ for 10 min immediately before image acquisition, as described above with a 4′,6-diamidino-2-phenylindole (DAPI) filter. Post-processing of images was performed with ImageJ software (version 1.54) [57] last accessed on 5 May 2023.

### 4.7. HEK-Blue hTLR4 Cell Treatments for Secreted Embryonic Alkaline Phosphatase (SEAP) Assay

HEK-Blue hTLR4 cells seeded in 24-well plates (Corning) were transfected with the same amount of pcDNA6.2/C-EmGFP-DEST plasmid encoding wild-type or mutated versions of IRAK3 genes, or empty plasmid (as a control), as described above. Cells were treated with 10 ng mL^−^^1^ lipopolysaccharide (LPS, *E. coli* O55:B5; Sigma-Aldrich) with or without 0.1 nM 8-bromoguanosine 3′,5′-cyclic monophosphate sodium salt (8-Br-cGMP; Merck), or an equal volume of vehicle (water) in DMEM medium supplemented with 8% (*w*/*v*) pre-heated FBS (30 min at 56 °C to inactivate AP activity) 24 h post-transfection. Cells were grown for 18–20 h at 37 °C in 5% CO_2_ tissue culture incubator. SEAP assay was performed according to manufacturer’s instruction in the HEK-Blue hTLR4 QUANTI-Blue solution protocol (InvivoGen). Briefly, 20 μL of cell supernatant was transferred in triplicate to a clear flat-bottom 96-well plate (Nunc) and 180 μL QUANTI-Blue solution was added. Upon incubation of the plate for 20 min at 37 °C, SEAP levels were determined spectrophotometrically at λ_655_ using the CLARIOstar Plus (BMG Labtech). Optical density data were normalized to eGFP fluorescence readout (λ_487_–λ_509_) of transfected cells to account for differences in IRAK3 protein expression levels due to variations in transfection efficiencies. Data were expressed as percentage change in LPS-induced NFκB activity upon data normalization to the control baseline (supernatant from LPS-treated cells transfected with empty vector, EV). Data from three independent experiments were analyzed (*p* value < 0.05; unpaired *t*-test with Welch correction).

### 4.8. HEK293T Cell Treatments for cGMP Generation and Quantification

HEK293T cells seeded in 6-well plates (Corning) were transfected with an equimolar amount of pcDNA6.2/C-EmGFP-DEST plasmid encoding wild-type or mutated versions of IRAK3 genes, or empty plasmid (as a control), as described above. GFP fluorescence (λ_487_–λ_509_) of transfected cells was measured with the CLARIOstar Plus 24 h post-transfection to account for differences in IRAK3 protein expression levels. Cells were treated with 2 mM MnCl_2_ for 10 min, upon which 0.5% (*w*/*v*) dodecyl trimethylammonium bromide supplied in the cGMP EIA system kit (GE Healthcare) was added to the medium and cells were agitated on a microplate shaker for 10 min to facilitate cell lysis. Cell lysates were used immediately in the immunoassay following protocol number 11 (‘New protocol 4. Total cellular cGMP measurement using novel lysis reagents for cell culture samples’) according to the manufacturer’s recommendations. Spectrophotometric measurements were performed at λ_405_ using the CLARIOstar Plus, were normalized to the eGFP fluorescence signal, and data from three independent experiments were analyzed (*p* value < 0.05; one-way ANOVA followed by Dunnett multiple comparison test).

### 4.9. Complementation of IRAK3 Knockout Cell Lines and Measurement of Cytokine Levels

Complementation of the THP-1 IRAK3^−/−^ knockout cell line (K6-3) with an empty pcDNA6.2/C-EmGFP-DEST plasmid (control) or with the plasmid encoding eGFP-tagged wild type IRAK3 or selected mutated versions of the protein (R97A, D377A, D385A, R97A/R372L) was performed as described previously [27]. Transiently transfected IRAK3 knockout cells were treated with 1 µg mL^−^^1^ LPS with or without 0.1 nM 8-Br-cGMP for 24 h. Levels of interleukin-6 (IL-6) and tumor necrosis factor-α (TNF-α) were measured using BD OptEIA IL-6 and OptEIA TNF-α ELISA kits (BD Biosciences) according to the manufacturer’s instructions. Data from 7–9 independent experiments were normalized to the eGFP fluorescence signal (excitation: 470–15 nm, emission: 515–20 nm) measured by CLARIOstar Plus and total protein concentration quantified by the Bradford method [58] and analyzed (*p* value < 0.05; one-way ANOVA followed by Turkey’s multiple comparisons test).

### 4.10. Prediction of Protein Associations and Protein—GTP Docking

The structure of pseudokinase domain IRAK3 (PDB 6RUU) [37] was retrieved from the RCSB Protein Data Bank (RCSB PDB; https://www.rcsb.org/ (accessed on 22 June 2021)) [59]. Protein–protein docking was performed using ClusPro (version 2.0; https://cluspro.bu.edu/ (accessed on 27 July 2021)) [60]. Docking of GTP to IRAK3 was performed using SwissDock (http://www.swissdock.ch/ (accessed on 9 December 2021)) [61]. The models and interactions were analyzed and visualized using UCSF Chimera (version 1.10.2) [62].

### 4.11. Statistical Analyses

Data were analyzed using GraphPad Prism 9 software (GraphPad Software) using the statistical tests indicated above. Intra-assay coefficient of variation (CV) was calculated within each plate and the average CV across all the plates for the n samples calculated. Inter-assay CV was calculated for each of the n samples across all plates. CV% was calculated by dividing the standard deviation by the mean and converting to a percentage (×100), and the overall CV was determined across the n samples for both intra- and inter-assay CVs.

## Data Availability

All data generated or analyzed during this study are reported in the manuscript and in the Supplementary Materials.

## Supplementary Materials

The following supporting information can be downloaded at: https://www.mdpi.com/article/10.3390/ijms24108572/s1.
