# Supplementary material for: Mutations in the Vicinity of the IRAK3 Guanylate Cyclase Center Impact Its Subcellular Localization and Ability to Modulate Inflammatory Signaling in Immortalized Cell Lines
Source: Int J Mol Sci. 2023 May 10;24(10):8572. doi: 10.3390/ijms24108572 (PMC10217915; doi:10.3390/ijms24108572)
Supplement: Supplementary file 1 [file ijms-24-08572-s001.zip › ijms-2340906-supplementary.pdf]

Supplementary information for

Mutations in the vicinity of the guanylate cyclase center of IRAK3 impact its subcellular localization and ability to modulate inflammatory signaling in immortalized cell lines

Ilona Turek [1,2,*], Trang H Nguyen [1,2], Charles Galea [3], Isaiah Abad [3], Lubna Freihat [3], David T. Manallack [3], Tony Velkov [4] and Helen Irving [1,2,3,*]

[1] Department of Rural Clinical Sciences, La Trobe Rural Health School, La Trobe University, Bendigo, Victoria 3552, Australia; i.turek@latrobe.edu.au, hongtrang.nguyen@latrobe.edu.au, h.irving@latrobe.edu.au

[2] La Trobe Institute for Molecular Science, La Trobe University, Bendigo, Victoria 3552, Australia

[3] Monash Institute of Pharmaceutical Sciences, Monash University, Melbourne, Victoria 3052, Australia; david.manallack@monash.edu

[4] Department of Microbiology, Monash University, Wellington Rd, Clayton VIC 3800, Australia; tony.velkov@monash.edu

* Correspondence: i.turek@latrobe.edu.au (I.T.); h.irving@latrobe.edu.au (H.R.I.); Tel.: +61354447551

The following pages include:

Supplementary Table S1: Sequences of PCR primers used for generating IRAK3 PD construct and for sequencing.

Supplementary Figure S1: Pseudokinase domain (PD) of IRAK3 contains guanylyl cyclase (GC) catalytic center and is sufficient to generate cGMP in vitro.

Supplementary Figure S2: Expression analyses of IRAK3 wild type and mutant proteins in baculovirus/insect cell system employing Spodoptera frugiperda (Sf9) and Trichoplusia ni (High Five) cells undertaken by the University of Queensland Protein Expression Facility (PEF).

Table S1. Sequences of PCR primers used for generating IRAK3 PD construct and for sequencing.

Name	Sequence (5′- 3′)
IRAK3 PD-For	GGGGCCAAGTTTGTACAAAAAAGCAGGCTCCATGCAGTGTAAGAAGCATTGG
IRAK3 PD-Rev	GGGGACCACTTTGTACAAGAAAGCTGGGTCCTAGCAGGGAGGCACTTTC
201-For	TCGCGTTAACGCTAGCATGGATCTC
IRAK3-For	GAACAAGAGAATTACTTTGGTCCTG
IRAK3-m-For	TGACAGATTGCAGTGTGTAG
IRAK3-Rev	CTCCAGGAATAGAGGAGAAGGA
GFP-Rev	TGGTGCAGATGAACTTCAGG

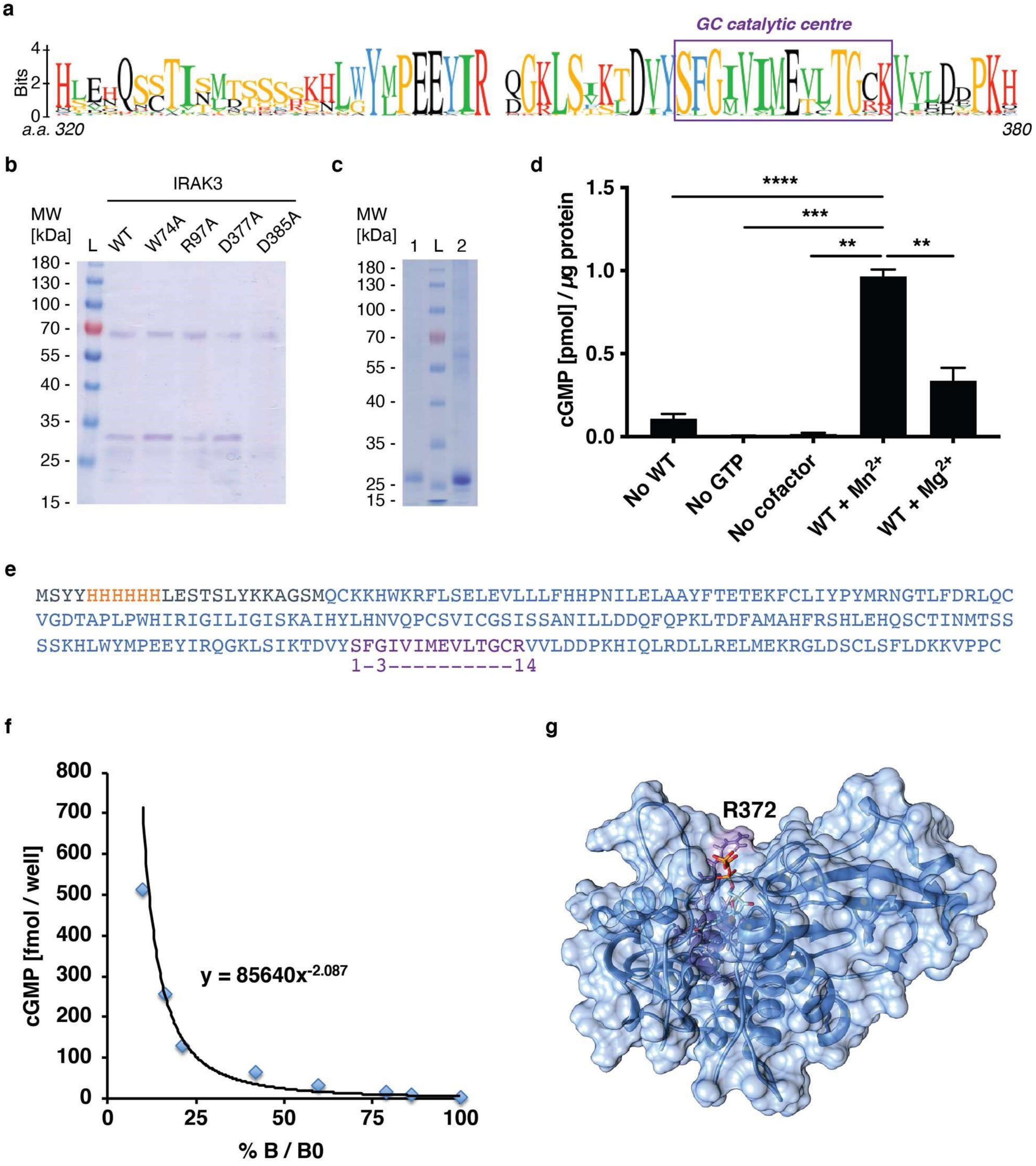

Figure S1. Pseudokinase domain (PD) of IRAK3 contains guanylyl cyclase (GC) catalytic center and is sufficient to generate cGMP in vitro.

(a) Consensus sequence of the portion of IRAK3 PD containing guanylyl cyclase (GC) catalytic center. The amino acid sequence logo was generated using a multiple sequence alignment of 165 IRAK3 orthologues in vertebrates. Amino acids are colored according to the Clustal coloring scheme (K, H, R in red; G, P, S, T in orange, F, W, Y in blue; I, L, M, V in green; D, E, C, Q, N, A in white). Amino acids are numbered according to the human IRAK3 (accession number: Q9Y616) sequence; (b) Coomassie brilliant blue-stained SDS-PAGE gel showing the purified full-length IRAK3 wild type (WT) and mutated recombinant proteins at the expected molecular weight (MW) of ~68 kDa. L—MW ladder (PageRuler Pre-

stained Protein Ladder, Thermo Fisher Scientific); (c) Coomassie brilliant blue-stained SDS-PAGE gel showing the purified recombinant IRAK3 containing a truncated pseudokinase domain (IRAK3 PD; amino acids 201 - 410) protein at the expected molecular weight (MW) of ~27 kDa from two separate clones 1 and 2. L—MW ladder (PageRuler Pre-stained Protein Ladder, Thermo Fisher Scientific); (d) full-length WT recombinant IRAK3 generates picomolar amounts of cGMP per µg protein in vitro, with a preference for Mn^{2+} over Mg^{2+} ions as a cofactor. Results from three independent experiments were plotted (mean ± SD, one-way ANOVA followed by Tukey–Kramer multiple comparison test, n = 3, ** $P < 0.01$, *** $P < 0.001$, **** $P < 0.0001$; intra-assay CV% = 9.3, inter-assay CV% = 2.4); (e) amino acid sequence of 6 x His-tagged (orange) recombinant IRAK3 containing truncated pseudokinase domain (PD; blue) with a 14 amino acid long guanylate cyclase (GC) catalytic center (purple) indicated, and residues predicted to be crucial for its enzymatic activity numbered; (f) exemplar standard curve for cGMP quantification, where cGMP content per well is expressed as a function of the percent bound (% B / B0); (g) docking of guanosine-5′triphosphate (GTP) at the GC catalytic center (in purple) of IRAK3 PD semi-transparent surface model. Docking of the GTP substrate (shown as a stick model) was performed automatically using SwissDock (http://www.swissdock.ch/). Residues comprising the GC catalytic center, including R372, are shown as stick models.

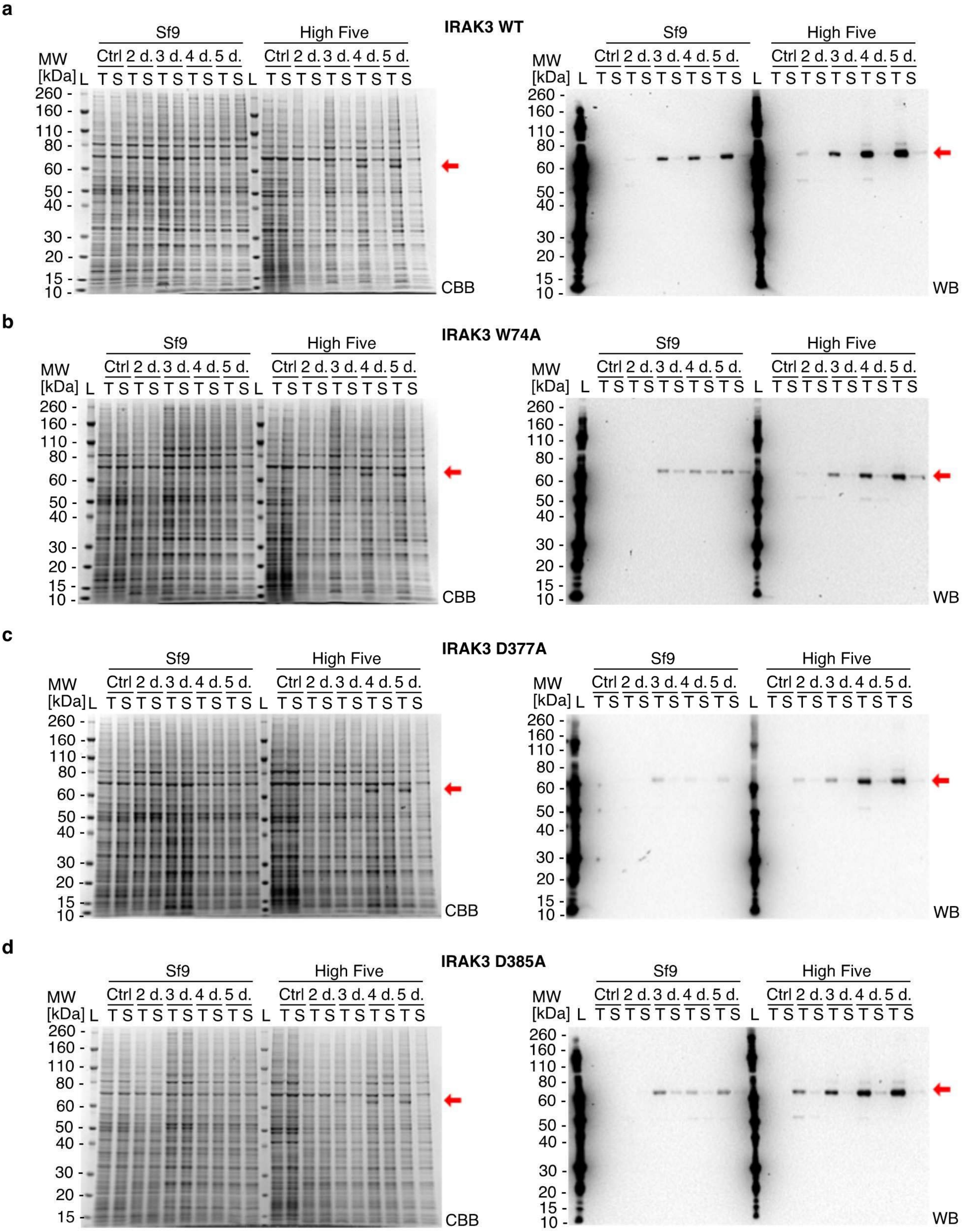

Figure S2. Expression analyses of IRAK3 wild type and mutant proteins in baculovirus/insect cell system employing Spodoptera frugiperda (Sf9) and Trichoplusia ni (High Five) cells undertaken by the University of Queensland Protein Expression Facility (PEF). Cells were grown at either 27□C and harvested 2 or 3 days post-inoculation, or at 21□C and

harvested 4 or 5 days post-inoculation. (a) Expression of wild type (WT) IRAK3; (b) expression of W74A IRAK3 mutant; (c) expression of D377A IRAK3 mutant; (d) expression of D385A IRAK3 mutant. Time-course samples analyzed by SDS-PAGE (4-12% Bis-Tris gel; left panel) stained with Coomassie brilliant blue (CBB) and Western blot (WB; anti-His–HRP (Miltenyi Biotec); right panel). IRAK3 proteins at the expected molecular weight (MW) of ~70 kDa are indicated with red arrows. T—total lysate; S—soluble fraction; Ctrl—control; L—MW ladder (Novex Sharp Pre-stain MW ladder); d. —day post-inoculation.
